# Transcriptome Analysis of Soursop (*Annona muricata* L.) Fruit under Postharvest Storage Identifies Genes Families Involved in Ripening

**DOI:** 10.3390/plants11141798

**Published:** 2022-07-07

**Authors:** Yolotzin Apatzingan Palomino-Hermosillo, Guillermo Berumen-Varela, Verónica Alhelí Ochoa-Jiménez, Rosendo Balois-Morales, José Orlando Jiménez-Zurita, Pedro Ulises Bautista-Rosales, Mónica Elizabeth Martínez-González, Graciela Guadalupe López-Guzmán, Moisés Alberto Cortés-Cruz, Luis Felipe Guzmán, Fernanda Cornejo-Granados, Luigui Gallardo-Becerra, Adrian Ochoa-Leyva, Iran Alia-Tejacal

**Affiliations:** 1 Unidad de Tecnología de Alimentos-Secretaría de Investigación y Posgrado, Universidad Autónoma de Nayarit, Ciudad de la Cultura SN, Tepic 63000, Nayarit, Mexico; pasingan@gmail.com (Y.A.P.-H.); veronica.ochoa@uan.edu.mx (V.A.O.-J.); balois_uanayar@hotmail.com (R.B.-M.); zurit_8@hotmail.com (J.O.J.-Z.); u_bautista@hotmail.com (P.U.B.-R.); mc.monica.martinez@gmail.com (M.E.M.-G.); 2 Unidad Académica de Agricultura, Universidad Autónoma de Nayarit, Carretera Tepic-Compostela, Km. 9, Xalisco 63780, Nayarit, Mexico; lguzman2303@hotmail.com; 3 Centro Nacional de Recursos Genéticos-Instituto Nacional de Investigaciones Forestales, Agrícolas y Pecuarias, Boulevard de la Biodiversidad 400, Tepatitlan de Morelos 47600, Jalisco, Mexico; cortescruz@hotmail.com (M.A.C.-C.); guzman.luisf@hotmail.com (L.F.G.); 4 Departamento de Microbiología Molecular, Instituto de Biotecnología, Universidad Nacional Autónoma de Mexico, Avenida Universidad 2001, Cuernavaca 62210, Morelos, Mexico; fernanda.cornejo@ibt.unam.mx (F.C.-G.); luigui.gallardo@ibt.unam.mx (L.G.-B.); adrian.ochoa@ibt.unam.mx (A.O.-L.); 5 Posgrado en Ciencias Agropecuarias y Desarrollo Rural, Universidad Autónoma del Estado de Morelos, Avenida Universidad 1001, Cuernavaca 62210, Morelos, Mexico; iran.alia@uaem.mx

**Keywords:** de novo assembly, differential gene expression, functional annotation, plant cell wall, refrigeration, pectin, softening

## Abstract

Soursop (*Annona muricata* L.) is climacteric fruit with a short ripening period and postharvest shelf life, leading to a rapid softening. In this study, transcriptome analysis of soursop fruits was performed to identify key gene families involved in ripening under postharvest storage conditions (Day 0, Day 3 stored at 28 ± 2 °C, Day 6 at 28 ± 2 °C, Day 3 at 15 ± 2 °C, Day 6 at 15 ± 2 °C, Day 9 at 15 ± 2 °C). The transcriptome analysis showed 224,074 transcripts assembled clustering into 95, 832 unigenes, of which 21, 494 had ORF. RNA-seq analysis showed the highest number of differentially expressed genes on Day 9 at 15 ± 2 °C with 9291 genes (4772 up-regulated and 4519 down-regulated), recording the highest logarithmic fold change in pectin-related genes. Enrichment analysis presented significantly represented GO terms and KEGG pathways associated with molecular function, metabolic process, catalytic activity, biological process terms, as well as biosynthesis of secondary metabolites, plant hormone signal, starch, and sucrose metabolism, plant–pathogen interaction, plant–hormone signal transduction, and MAPK-signaling pathways, among others. Network analysis revealed that pectinesterase genes directly regulate the loss of firmness in fruits stored at 15 ± 2 °C.

## 1. Introduction

Soursop (*Annona muricata* L.) is a member of the Annonaceae family that is cultivated in tropical and subtropical countries such as Mexico, Venezuela, Ecuador, Colombia, and Brazil [1,2]. Mexico is the top producer of this crop worldwide and Compostela, Nayarit represents approximately 81% of the total production at a national level, with an annual income of 12.2 millions of dollars [3,4]. The fruits are mainly consumed fresh and the pulp can be used to develop several products such as ice-cream, juice, nectar, jelly, yogurt, among others [2]. Furthermore, in the last years there has been an increase in the interest of this crop due to its medicinal properties such as anthelmintic, antihypertensive, anti-inflammatory, antidiarrhea, antiparasitic, antimalarial, anticancer activities, among others [5]. Soursop is a climacteric fruit with a high respiration rate and ethylene production causing a fast fruit softening, leading to postharvest decay [6,7,8]. The peak of ethylene production of soursop is between five and six days of storage, which seems to be associated with the activation of several enzymes [7,9]. Fruit ripening is a complex process that involves modifications in the cell wall and secondary metabolism, leading to changes in flavor, texture, and aroma, among others [10]. The loss of firmness and textural changes is related to the action of cell wall enzymes such as polygalacturonase (*PG*), pectinesterase (*PME*), pectate lyase (*PL*), glucosidase (*Glu*), expansin (*EXP*), among others [11,12]. Nonetheless, the molecular mechanism of this process remains unclear. The short postharvest shelf life of soursop is one of the main factors limiting exportation to international markets. Therefore, some methods have been studied to maintain the quality and delay fruit ripening of soursops such as refrigeration, waxes, emulsions, edible coatings, 1-methylcyclopropene, and the combinations of all these [6]. Among them, refrigeration is the most simple, efficient, and commonly used postharvest technology utilized in fruit conservation [7,9,13]. Some authors have shown that soursop fruits stored at 15 ± 2 °C delay ripening and prolong the shelf life of the fruit up to 7 or 8 days without physiological damage [7,14]. However, few studies have been focused on the changes during postharvest storage of soursop fruit at the molecular level. In this regard, our research group has identified the reference and differentially expressed genes by quantitative real-time polymerase chain reaction (qRT-PCR) during postharvest storage [11,15,16]. Nevertheless, the regulatory mechanism of this process has not been fully elucidated due to the lack of transcriptomic resources. Transcriptomic (RNA-seq) analysis is a sequencing technology that has the potential to unravel the metabolic pathways, action mechanisms and identify the differences in gene expression levels of an organism under certain conditions [17]. Moreover, few transcriptomics of the Annonaceae family have been analyzed. RNA-seq analysis has been used in flowers and fruits of sugar apple (*Annona squamosa* L.), identifying several genes involved in floral development and pathways related to primary, secondary metabolism, seed and fruit development expressed, respectively [18,19,20]. To the best of our knowledge, only sequence data without annotation of soursop leaves have been generated [6,21] and no soursop fruit transcriptome can be found.

To better understand the mechanisms involved in fruit ripening, the objective of this study was to analyze the soursop fruit transcriptome under different postharvest storage conditions. This study provides transcriptome data of the soursop fruits and gives important information about the genes and mechanisms that are being regulated.

## 2. Results

### 2.1. Physicochemical Analysis

**Figure 1:**
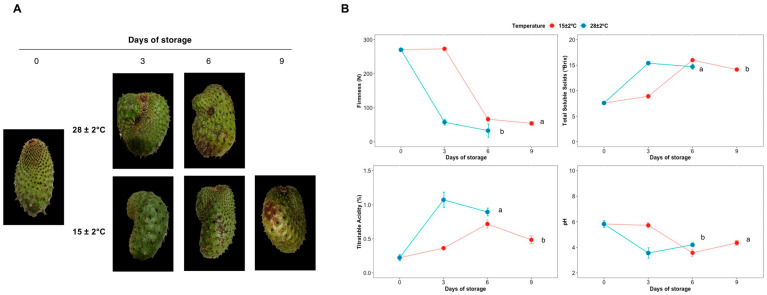
Experimental strategy and physicochemical analysis. (**A**) Soursop fruits stored at 28 ± 2 °C and 15 ± 2 °C (days of storage are the sample points after storage). (**B**) Firmness, Total soluble solids, Titratable acidity, and pH of soursop fruits. The points represent the mean of nine measurements and the vertical lines indicate the standard deviation of the means. Different letters indicate statistically significant differences at *p* < 0.05 between temperatures using the means of 27 and 36 data per the temperature stored at 28 ± 2 °C and 15 ± 2 °C, respectively.

The experimental strategy consisted of fruits stored at 28 ± 2 °C and 15 ± 2 °C on different days of storage, as shown in Figure 1A. The effect of temperature on firmness, total soluble solids, titratable acidity, and pH was determined (Figure 1B). The soursop fruits stored at 28 ± 2 °C showed a dramatical decrease of firmness from Day 0 to Day 6 at 28 ± 2 °C, losing 87.95% of firmness. Otherwise, the fruits stored at 15 ± 2 °C reached a mean value of 53.7 N up to Day 9 of storage, indicating a loss of firmness percentage of 80.14% compared to Day 0 (Figure 1B). The temperature of 15 ± 2 °C delays the rate of loss of firmness in the soursop fruit compared to fruits stored at 28 ± 2 °C (*p* < 0.05). The total soluble solids and titratable acidity increased from Day 0 to Day 3, followed by a decrease on Day 6 in the fruits stored at 28 ± 2 °C. This same behavior was observed in fruits stored at 15 ± 2 °C from Day 3 to Day 9. Nevertheless, the temperature of 28 ± 2 °C showed significant changes in comparison with the fruits stored at 15 ± 2 °C (*p* < 0.05). On the contrary, pH decreased from Day 0 to Day 3 followed by an increase on Day 6. Indeed, the fruits stored at 15 ± 2 °C showed the same behavior from Day 3 to Day 9. Moreover, the fruits stored at 15 ± 2 °C presented significant differences in pH compared to those stored at 28 ± 2 °C. The same conditions were used for RNA extraction, cDNA libraries construction, and sequencing.

### 2.2. De Novo Assembly and Functional Annotation of Soursop Fruit Transcriptome

RNA-seq libraries were constructed using RNA from different days and temperature conditions of soursop fruits. In total, 170.38 GB of raw reads were obtained. Low-quality reads and adapters were removed (Q < 20) and clean reads were used for the *de novo* transcriptome assembly. The final assembly generated 224,074 transcripts (445.2 MB) with a median contig length of 928 bp, average contig length of 1554.49, N50 value of 2839, and average GC percent content of 41.18%, clustering into 95,832 unigenes (90 MB) of which 21,494 had ORF. The stats of these unigenes were median contig length of 407 bp, average contig length of 915.70, and N50 of 1971 bp. For the functional annotation, the *de novo* assembled transcripts were queried (with BLAST) against several databases using the Trinotate annotation protocol. The Trinotate annotation results are shown in Table 1. Homology searches using BLASTx against the UniProt/SwissProt and Uniref90 databases were able to annotate 104,280 and 132,770 unique sequences, respectively. Indeed, BLASTp annotation against UniProt/SwissProt and Uniref90 databases found 94,538 and 136,059 unique proteins, respectively (Table 1). Moreover, unique GO terms using BLASTx and BLASTp were 11,829 and 13,307, respectively. The number of hits of candidate coding regions against other databases, including Pfam, SignalP, TmHMM, KEGG, and eggNOG, is also shown in Table 1.

**Figure 2:**
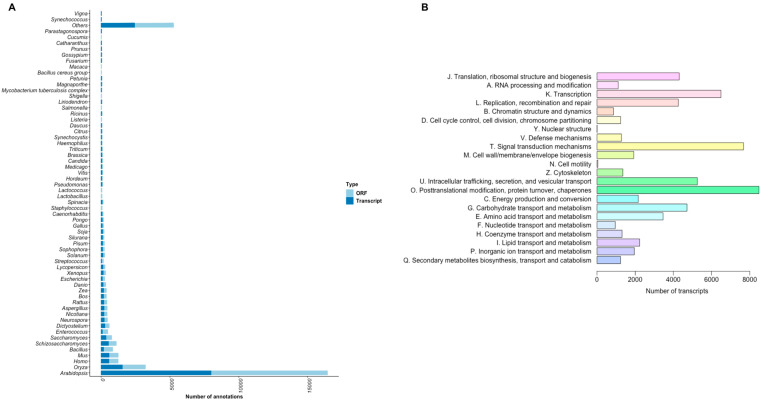
Functional annotation using BLASTx, (**A**) Top-hit genus taxonomic distribution (ORF and transcripts). (**B**) Number of transcripts matching with the eggNOG database. Different color represents the pathways categorized in information storage and processing (J, A, K, L, B), cellular processes and signaling (D, Y, V, T, M, N, Z, U, O), and metabolism (C, G, E, F, H, I, P, Q).

On the other hand, the number of unique genes, transcripts, and proteins with Pfam annotation was 22,319 (23.28% of all genes), 94,352 (42.10% of all transcripts), and 125,655, respectively. The BLAST top annotation hits distribution showed that the highest number of ORF and transcripts were for the genus *Arabidopsis* followed by *Oryza*, as shown in Figure 2A. Based on sequence homology against the eggNOG database, soursop fruit genes were categorized into three main groups: information storage and processing (J, A, K, L, B), cellular processes and signaling (D, Y, V, T, M, N, Z, U, O), and metabolism (C, G, E, F, H, I, P, Q), finding the highest number of transcript in posttranslational modifications followed by signal transduction mechanisms, corresponding to the cellular processes and signaling as shown in Figure 2B.

**Figure 3:**
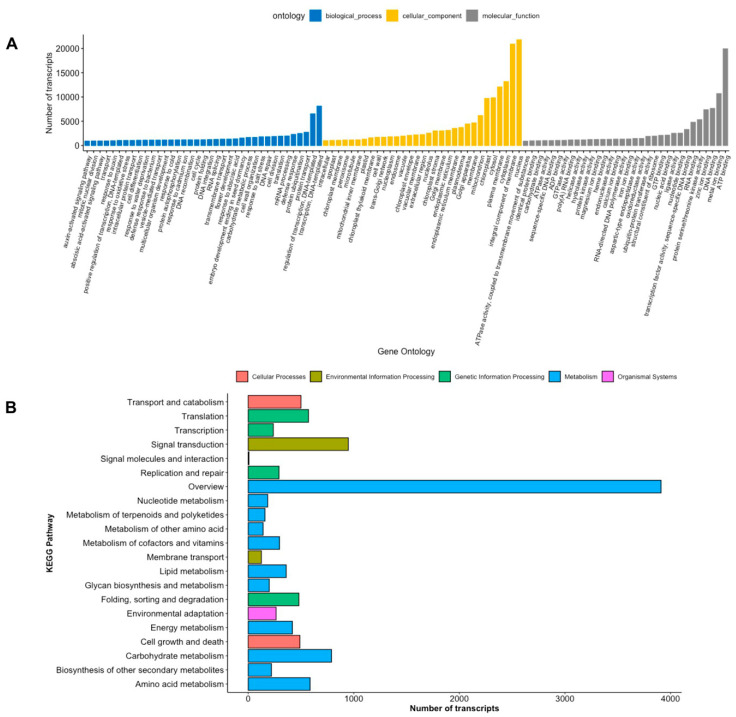
Gene ontology and KEGG pathways annotation. (**A**) Number of transcripts by GO category. (**B**) Number of transcripts by KEGG pathways. Different colors represent the GO category and KEGG pathways.

Further, the soursop transcripts were classified into three GO categories: Biological process, cellular component, and molecular function, as shown in Figure 3A. Under the biological process category, transcription, DNA-templated represented the highest number of transcripts with 8113 transcripts (1770 unigenes). Within the cellular component category, the nucleus showed 21,790 transcripts (4734 unigenes). ATP-binding was highly represented in the molecular function category, with 19,925 transcripts (4575 unigenes). Likewise, genes were also analyzed in the KEGG database, finding that the most significant pathways regarding the number of hits were related to overview and carbohydrates of the Metabolism type, with 3910 genes and 789 genes, respectively (Figure 3B). On genetic information processing type, 238 genes were detected in the transcription pathway, and in environmental information processing, 125 genes were involved in the membrane transport pathway. Transport and catabolism showed 500 genes related to the cellular process and 264 in environmental adaptation to the organismal system as shown in Figure 3B. Focusing on the pathways associated with ripening, according to the eggNOG database, 1996 transcripts code for proteins located in the cell wall or in the membrane (Figure 2B). Further, transcripts related to the cell wall were found in the ontology of biological process (470 genes) and cellular components (712 genes), as well as cell growth and death (Figure 3A,B).

### 2.3. DEG in Response to Postharvest Storage

**Figure 4:**
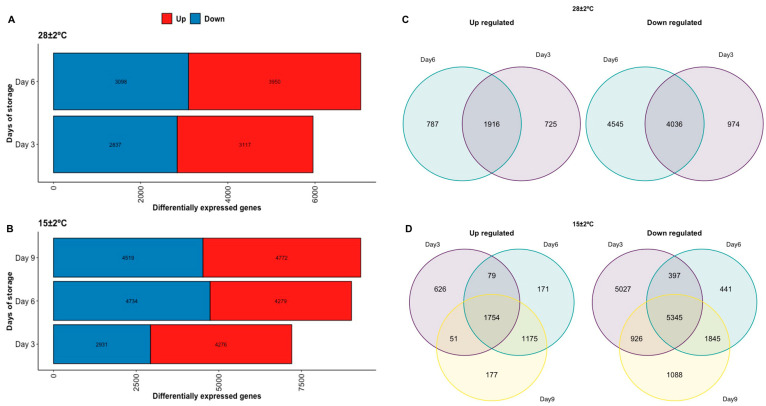
DEG under different temperatures (**A**) Up and down-regulated genes per day of storage at 28 ± 2 °C. (**B**) Up and down-regulated genes per day of storage at 15 ± 2 °C. (**C**) Venn Diagram of shared up and down-regulated per day of storage at 28 ± 2 °C. (**D**) Venn Diagram of shared up and down-regulated per day of storage at 15 ± 2 °C.

Annotated DEG were used to develop an online public database called Annomics, which can be freely http://perseo.uan.mx/bioinformatica/annomicsdatabase (accessed on 5 July 2022). The number of DEG (up and down-regulated) by each pairwise comparison against Day 0 is shown in Figure 4A,B. The Day of storage 9 followed by Day 6 at 15 °C ± 2 °C presented the highest DEG (up and down), as shown in Figure 4B. Day 9 at 15 °C ± 2 °C presented 9291 DEG, including 4772 up and 4519 down-regulated genes and Day 6 at 15 °C ± 2 °C displayed 9013 DEG, involving 4279 up and 4734 down-regulated genes. Furthermore, the Venn diagram revealed that the number of down-regulated genes shared between the days of storage was higher than the up-regulated in both temperatures (Figure 4C,D). The temperature of 28 ± 2 °C showed a higher number of up-regulated genes shared between the days of storage compared to the temperature of 15 ± 2 °C (Figure 4C,D). On the other hand, the temperature of 15 ± 2 °C presented a higher number of down-regulated genes shared by the days of storage than the temperature of 28 ± 2 °C.

**Figure 5:**
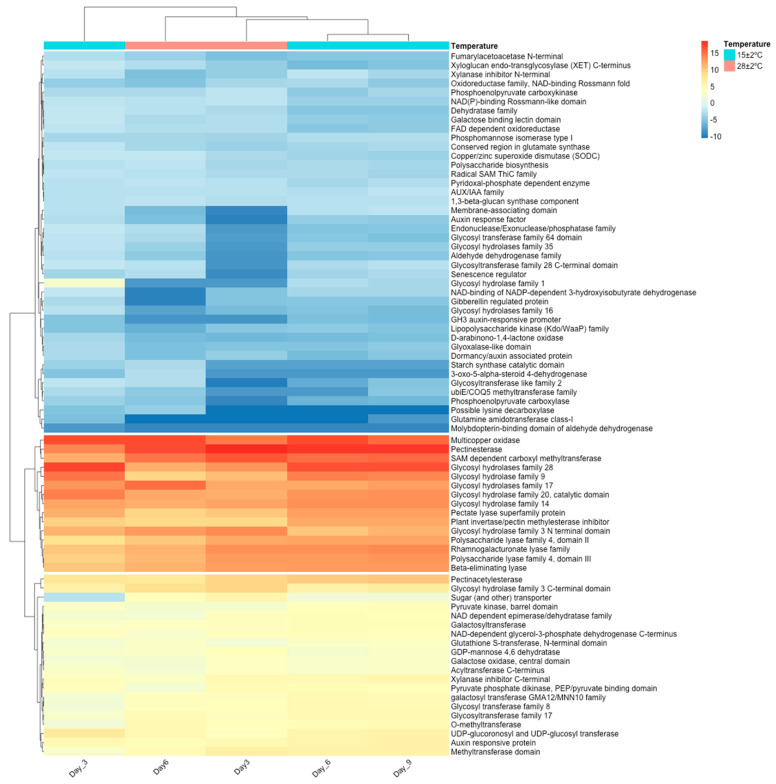
Heatmap of the DEG associated with ripening per day of storage at 28 ± 2 °C and 15 ± 2 °C. The dendrogram shows the relationship between gene expression by hierarchical clustering. The color key indicates the LogFC of the DEG (FDR < 0.001), ranging from blue to red.

Based on the functional annotation by BLASTx, a heatmap with hierarchical clustering analysis of the DEG associated with ripening was performed using the highest LogFC per selected gene family (Figure 5). The cluster analysis showed three clusters (k = 3) formed on low, high, and middle LogFC, respectively. In this context, the group with the highest LogFC was found in DEG involved mainly in the degradation of pectin, a principal component of the plant cell wall, followed by the group with middle LogFC comprising DEG related with starch and sugar metabolism, carbohydrates metabolic process, and hydrolase activity, among others (Figure 5).

Finally, the DEG group with the lowest LogFC is involved in several pathways such as hormone regulation, oxidation, among others. The pectinesterase gene showed the highest LogFC in all the temperatures and days of storage evaluated. Indeed, functional enrichment analysis showed more than 10 significant (corrected *p*-value < 0.05) GO terms related to ripening, including GO:0030599, which is associated with the pectinesterase activity (Table 2). The GO annotation of these terms was related with cell wall modification and organization, enzyme activity (pectinesterase and polygalacturonase), hydrolase activity, and polysaccharide activity, as shown in Table 2.

**Figure 6:**
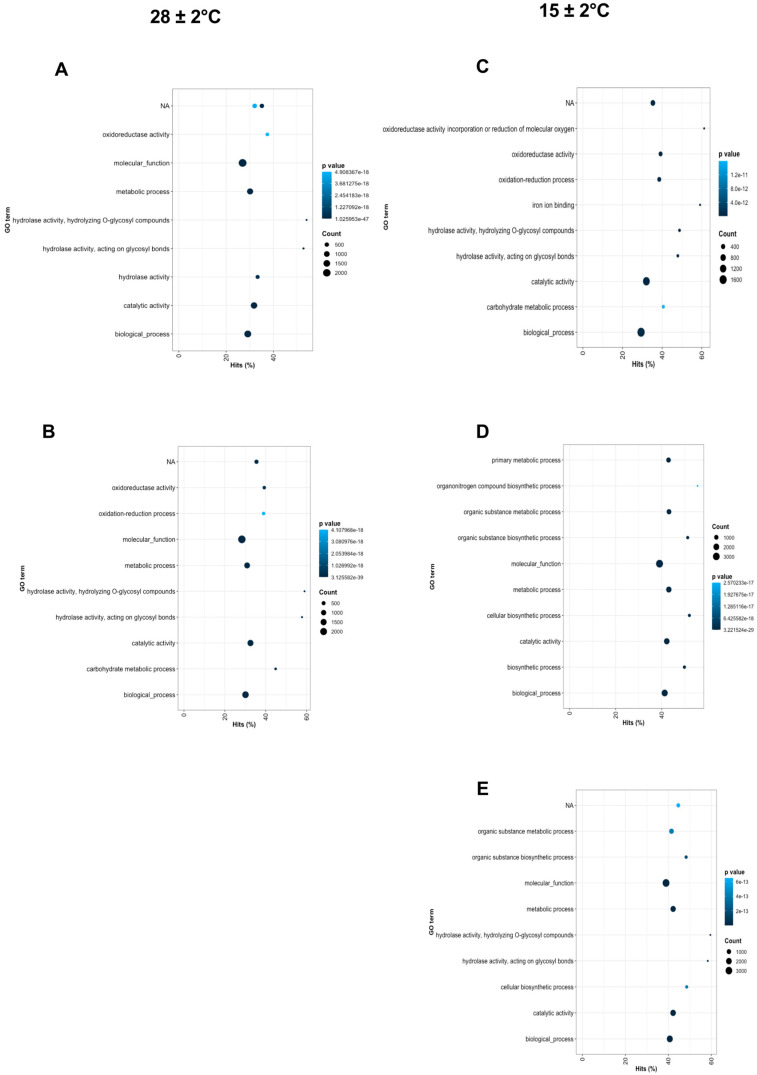
Top 10 significantly enriched GO terms of the DEG by each pairwise comparison against Day 0, (**A**) Day 3 at 28 ± 2 °C, (**B**) Day 6 at 28 ± 2 °C, (**C**) Day 3 at 15 ± 2 °C, (**D**) Day 6 at 15 ± 2 °C, (**E**) Day 9 at 15 ± 2 °C. The *y*-axis represents the GO terms, and the *x*-axis represents the percentage hits of enriched genes. The circle size indicates the genes in each GO term. Color scale means adjusted *p*-value.

**Figure 7:**
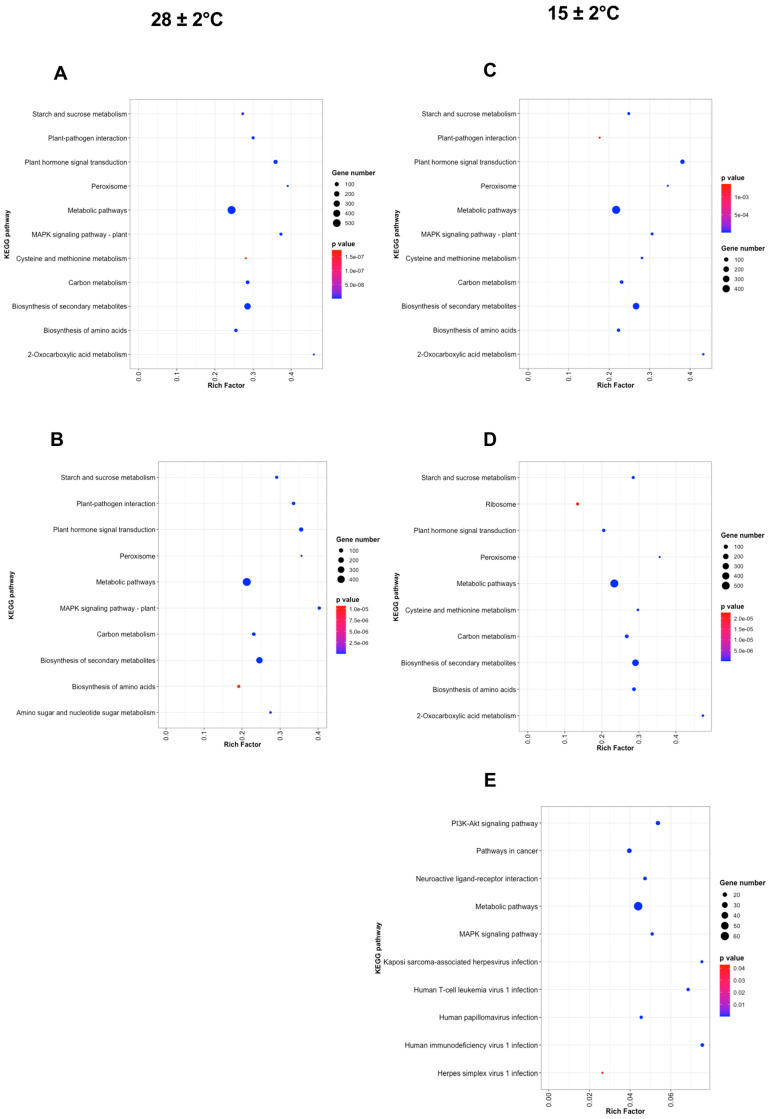
Top 10 significantly enriched KEGG pathways of the DEG by each pairwise comparison against Day 0, (**A**) Day 3 at 28 ± 2 °C, (**B**) Day 6 at 28 ± 2 °C, (**C**) Day 3 at 15 ± 2 °C, (**D**) Day 6 at 15 ± 2 °C, (**E**) Day 9 at 15 ± 2 °C. The *y*-axis represents the KEGG enriched pathways and the *x*-axis represents the Rich Factor. The circle size indicates the number of genes in each KEGG pathway. Colors correspond to the adjusted *p*-value.

We performed a GO biological process and KEGG pathway enrichment analysis of the DEG by each pairwise comparison and the top 10 were plotted, as shown in Figure 6 and Figure 7. Among the GO terms, the most DEG were significantly enriched in biological process and catalytic activity between all conditions (Figure 6A–E). Indeed, molecular function and metabolic process also had significant enrichment between all conditions except for Day 3 at 15 ± 2 °C (Figure 6C). According to the Rich Factor of the KEGG pathway, metabolic pathways, biosynthesis of secondary metabolites, plant hormone signal, starch, and sucrose metabolism, plant–pathogen interaction, plant–hormone signal transduction, and MAPK-signaling pathway were functionally enriched between Day 3 at 28 ± 2 °C–Day 6 at 15 ± 2 °C against Day 0 (Figure 7A–D). The high number of DEG were enriched in the metabolic pathway followed by biosynthesis of secondary metabolites and starch sucrose metabolism (Figure 7A–D). Metabolic and MAKP-signal pathways were enriched in all the conditions evaluated (Figure 7A–E). In the case of Day 9 at 15 ± 2 °C, most of the DEG were enriched in pathways related to human diseases (Figure 7E).

### 2.4. Correlation and Gene Expression Networks

**Figure 8:**
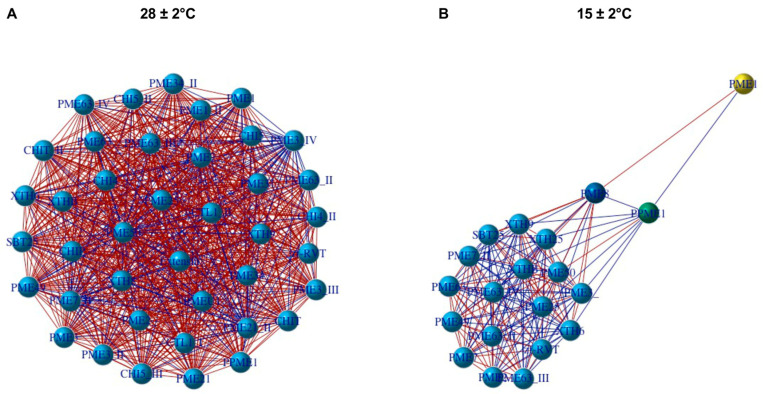
Network analysis of genes related to ripening (**A**) Genes at 28 ± 2 °C and (**B**) Genes at 15 ± 2 °C. Red lines mean high correlation while blue means low correlation.

Due to the DEG with the highest LogFC being related to degradation of pectin, we used the GO terms related to the plant cell (Table 2) to perform a correlation analysis between the firmness and enriched DEG. We found 20 and 40 unique enriched DEG at 15 ± 2 °C and 28 ± 2 °C, respectively. The highest correlation genes with the firmness (r > 0.05 and *p* < 0.05) were xyloglucans endotransglycosylase and pectinesterase at both temperatures. Additionally, chitinase genes showed a high correlation for the temperature of 28 ± 2 °C. On the other hand, the gene network analysis showed positive (red color of edges) and negative (blue color of the edges) interaction between the enriched DEG, forming a complex expression network at 28 ± 2 °C (Figure 8A). Interestingly, the gene expression network at 15 ± 2 °C showed a key Pectinestarese (PMEI) gene-regulating this process (Figure 8B). Further, PMEI presented a direct relationship with another two Pectinase genes, PME8 (high correlation) and PME1 (low correlation). These genes (PME8 and PME1) are interacting (positive and negative) with the other enriched DEG genes (Figure 8B). This indicates that pectinesterase genes directly regulate the loss of firmness in the fruit at 15 ± 2 °C.

### 2.5. RNA-Seq Data Validated through qRT-PCR

**Figure 9:**
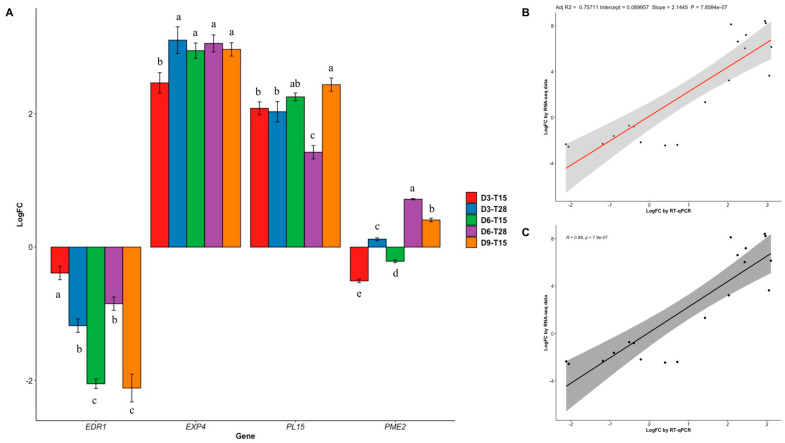
Validation of RNA-seq results using qRT-PCR. (**A**) Gene expression of four genes. (**B**) Linear regression. (**C**) Correlation. Different letters per gene indicate a significant difference (*p* < 0.05).

To validate the RNA-seq data, four unigenes with different expression patterns (up and down-regulated genes) were selected for qRT-PCR analysis under all the conditions evaluated. These genes were involved in plant cell wall metabolisms such as ethylene-responsive protein kinase (*EDR1*), Expansine (*EXP4*), pectate lyase (*PL15*), and pectinesterase (*PME2*). The LogFC results in all conditions are shown in Figure 9A. Considering the normalization of gene expression with Day 0 as one, the LogFC showed that the *EDR1* gene was down-regulated in all conditions tested, presenting Day 6 and Day 9 at 15 ± 2 °C with the highest down-regulation values (*p* < 0.05) compared to the other conditions. On the other hand, *EXP4* and *PL15* genes were up-regulated in all the conditions evaluated. Furthermore, Day 6 and Day 9 at 15 ± 2 °C significantly promoted (*p* < 0.05) the up-regulation of the *PL15* gene. Finally, in the *PME2* gene, considerable (*p* < 0.05) up-regulation was recorded on Day 3 and Day 6 at 15 ± 2 °C and down-regulation on Day 6 at 28 ± 2 °C and Day 3 at 15 ± 2 °C, respectively. The selected genes had the same pattern between the RNA-seq and qRT-PCR results (Supplementary Material Table S1). Linear regression analysis and correlation analysis between the LogFC data from RNA-seq and qRT-PCR showed a coefficient of R^2^ = 0.75711 (Figure 9B) and R = 0.88 (Figure 9C) with a *p*-value <0.01. Therefore, qRT-PCR results were consistent with the expression profiles found by the RNA-seq, considering a good validation of the DEG.

## 3. Discussion

Soursop fruit suffers rapid senescence after harvest, leading to several physiological changes at room temperature. The temperature of 15 ± 2 °C delayed ripening, formation of soluble solids, acidity, and pH without chilling injury, increasing the postharvest shelf life of the soursop fruits by three days compared with the fruits stored at 28 ± 2 °C. Previous studies have shown that refrigeration technology prolongs the postharvest shelf life of the fruit without causing cold damage [7,14]. On the other hand, approximately 90% of the soursop fruit production is consumed at a national level, however, it is not evenly distributed around Mexico and is only available in two seasons per year. [4]. Therefore, the stored temperature at 15 ± 2 °C will increase the accessibility to this fruit by transporting the fruit by truck, ship, or plane, reaching other regions of Mexico and, in the near future, this will favor the commercialization to other countries such as the USA, which is approximately 1400–1500 km distance by road from Nayarit to Arizona. The information about how genes and mechanisms are being regulated in soursop fruits during ripening at postharvest storage is practically null. Here, we used RNA-seq analysis and reported global transcriptional changes as well as pathways associated with ripening. The assembly statistics of this study showed a higher number of assembled transcripts and unigenes compared to other Annonaceae transcriptomes [19,20,21]. Recently, the first soursop genome was assembled at a chromosome level from PacBio and Illumina short-reads, identifying 23,375 protein-coding genes using de novo RNA-seq and homology searches [22]. In this study, similar results were obtained, finding 21,494 ORF from 95,832 unigenes under different postharvest storage conditions. On the other hand, the functional annotation showed genes related to pathways highlighting, transcription, signal transduction, and metabolism that respond to different postharvest storage conditions. Taken together, the transcriptomic results presented here expand the knowledge of soursop fruit.

We identified several DEG in soursop fruit at 28 ± 2 °C and 15 ± 2 °C on different days of storage, allowing us to gain insights into the gene regulation during postharvest storage in soursop fruits. Within this information, we created the first public database of DEG in soursop fruit under different postharvest storage conditions. In this regard, 14,701 datasets with unique annotation were fully uploaded, including its sequence, symbol, Pfam, and annotation http://perseo.uan.mx/bioinformatica/annomicsdatabase (accessed on 5 July 2022). The temperature of 15 ± 2 °C showed the highest number of up-regulated genes. Indeed, the number of DEG increased according to the days and temperature storage conditions. In this context, the largest number of DEG, as well as up-regulated genes, was recorded after nine days of storage at 15 ± 2 °C, representing 10.31% of the total genes assembled in this study. A possible explanation for this result is that the temperature has a direct impact on plant growth and development due to the structure and composition of the cell wall can change. Moreover, this condition coincides with the onset of senescence, leading to the accumulation of secondary metabolites and cell wall breakdown, involving several genes.

Transcriptomic studies applying low storage temperatures (below 15 °C) showed the highest DEG in different fruits and vegetables [23,24]. These results suggest that the regulation of gene expression is mediated by the combination of days and temperature of postharvest storage. On the other hand, Day 6 of storage at the temperature of 28 ± 2 °C showed the highest number of DE and up-regulated genes. This indicates that more genes are induced during the maturity of consumption than physiological maturity, which directly impacts the soursop fruit softening, an important characteristic of the ripening. Similar results were obtained in mango fruits using RNA-seq technology [10].

The maturation involves several enzymes associated with the pectin, a polysaccharide that plays a critical role in the plant cell wall architecture. Remarkably, the top DEG were related to pectin, showing all these DEG high expression in all the conditions evaluated. Methyltransferases (S-adenosyl-L-methionine-dependent-methyltransferases) are a large plant family that contain enzymes that methylate the oxygen atom of several secondary metabolites such as phenylpropanoids, flavonoids, and some alkaloids, playing an important function in lignin biosynthesis [25]. Otherwise, *PE* plays a key role in cell wall metabolism during fruit ripening at the early stages of softening, specifically in the assembly and disassembly of the pectin [26]. It has been detected in soursop pulp, being one of the most heat-resistant enzymes [27,28]. On the other hand, enzymes such as glycosyl hydrolases, lyases, and glycosidases catalyze the degradation of polysaccharide connection [29]. In this context, we found Glycohydrolase highly expressed in all the conditions evaluated. Additionally, other cell wall enzymes located in the pectin such as *PL* and Rhamnogalacturonan lyase (*RGL*) were highly expressed. These are classified as pectin degrading enzymes and are implicated in fruit softening [30,31]. Tomato and strawberry have been used as plant models to study fruit ripening by using genetically modified plants. The results of that studies have demonstrated that *PL* and *RGL* contribute to fruit softening [32,33,34,35,36]. The ripening process is regulated by a diverse number of genes that control softening, the accumulation of sugars, and acids, among others [37]. In our study, we found that metabolic, biosynthesis of secondary metabolites, plant hormone signal, starch and sucrose metabolism, plant–pathogen interaction, plant–hormone signal transduction, and MAPK-signaling pathways were functionally enriched in most of the conditions evaluated. These KEGG pathways are closely related to the changes that occur during fruit softening, impacting the soursop characteristics such as color, firmness, taste, and flavor. Indeed, based on enriched DEG, we found a high correlation between firmness and pectin-associated genes. Previous studies on soursop fruits have demonstrated a negative correlation between the PME activity and the firmness of the fruits [7]. The pectinesterase gene family has several members with distinct biological roles, leading to a different function in the plant cell wall. Therefore, some pectinesterase genes are positive and others are negatively correlated with firmness. Moreover, the gene co-expression network at 15 ± 2 °C showed a key pectinesterase gene interacting with another two pectinesterase from the same family.

Collectively, these results indicate that multiple gene families associated with pectin, such as pectinesterase, xyloglucanase, Glycohydrolase, and rhamnogalacturonan lyase, among others, are involved in cell wall composition, directly impacting the loss of firmness of soursop fruits. Further, some members of the xyloglucans endotransglycosylase families have demonstrated differential expression under stress conditions, leading to delayed growth of the cell due to the increase of ROS [38].

Taking all these results, it seems that the temperature of 15 ± 2 °C modifies the structure of the plant cell wall, activating multiple cell wall-related genes depending on the development stage. Further, our results suggest that a set of pectin-associated genes are regulating these complex reactions.

We validate the RNA-seq results using qRT-PCR by analyzing the expression of up and down-regulated genes related to plant cell wall that impacts fruit softening. We demonstrated that our results were reliable due to the good linear and correlation coefficient values reported. Fruit ripening involves several genetic, biochemical, and physiological changes which are caused by a range of modifications in the polymers of the plant cell wall [39]. The plant hormone ethylene regulates a variety of physiological processes including fruit ripening. Ethylene is sensed by several receptors that, together with the Raf-like kinase constitutive triple response (*CTR1*), negatively regulate the ethylene signaling transduction [40,41]. In accordance, *EDR1* expression showed a down-regulation in all the conditions evaluated, suggesting that other receptors may exist for modulation of the ethylene in soursop fruit.

Pectin is solubilized in fleshy fruits, increasing the content of pectin, and causing the cell wall to dissemble in the plants [39]. Further, the most important pectin degrading enzymes are *PG*, *EXP*, *PME*, and *PL*, which are associated with fruit softening [31]. We found that in the fruits stored at 15 ± 2 °C, higher expression was recorded at nine days of storage compared to day three in the *EXP4*, *PL15*, and *PME2* genes. These results suggest that the combination of temperature and the onset of ripening induced the pectin degrading enzymes evaluated during postharvest storage.

## 4. Materials and Methods

### 4.1. Plant Material

Soursop fruits ‘GUANAY-1′ were hand-harvested at physiological maturity according to fruit shape, peel color, and size from 10 ungrafted trees in a 23 year-old orchard located in Venustiano Carranza, Nayarit, Mexico (21°32′2.77′′ N, 104°58′39.73′′ W) as reported by [15]. Five fruits per tree (50 fruits in total) without mechanical and pathogenic damage were selected, disinfected with 2.0% sodium hypochlorite, and washed with distilled water. Soursop fruits were stored at 28 ± 2 °C and 15 ± 2 °C in a controlled temperature chamber (Climacell^®^ CLC-B2V-M/CLC404-TV, Angelbachtal, Germany) until reaching senescence. Fruit mesocarp was collected in three developmental stages: physiological maturity (0 days), maturity of consumption (3 days at 28 ± 2 °C), and onset of senescence (6 days at 28 ± 2 °C). Further, the postharvest shelf life was prolonged up to 9 days at 15 ± 2 °C (onset of senescence). Hence, fruit mesocarp was taken at 0, 3, 6, and 9 days at 15 ± 2 °C as reported by [15]. The samples were quickly placed in RNAlater solution (Sigma-Aldrich), frozen in liquid nitrogen, and stored at −80 °C.

### 4.2. Physicochemical Analysis

Five fruits per each condition tested were used to measure the following physicochemical parameters: firmness, total soluble solids, titratable acidity, and pH. Firmness was measured with a digital penetrometer (SSEYL GY-4 Digital Fruit Penetrometer) in three different areas of fruit and reported as Newtons (N). The pH of the pulp was measured with a potentiometer (Hanna Instruments HI2210). The total soluble solids (TSS) were determined using a digital refractometer (Hanna HI 96801). Titratable acidity was determined according to the official method of the [42] by volumetric titration with 0.01 N of NaOH and phenolphthalein as an indicator.

### 4.3. RNA Extraction and RNA-seq Library Construction

Total RNA was extracted from 75 mg of soursop mesocarp tissue with the Spectrum Plant Total RNA kit (Sigma-Aldrich) following the manufacturer’s instructions. The RNA extracted was treated independently with the NEBNext Ultra RNA Library Prep Kit for Illumina Kit (New England BioLabs, Ipswich, MA, USA, Cat.E7530S). We used 700 ng of RNA as input for each library, adjusted the size select conditions for average insert sizes of 400 bp, and the enrichment PCR to 15 cycles. The quantity and quality of all libraries were assessed by Qubit Fluorometer (Invitrogen, Waltham, MA, USA, Cat.Q32851) and Agilent 2100 Bioanalyzer (Agilent Technologies, Santa Clara, CA, USA, Cat. 5067-4626), respectively. Good quality RNA was used to create independent RNA-seq libraries per each condition evaluated. The libraries were sequenced using the Illumina Next-Seq 500 High Output in a 300-cycle paired-end format at the National Institute of Genomic Medicine (INMEGEN) in Mexico City, Mexico.

### 4.4. De Novo Transcriptome Assembly

First, the quality visualization was performed with FastQC (version 0.11.5) [43]. After, we used Trimmomatic (version 0.36) [44] to remove the sequencing adapters, ambiguous nucleotides, and filter the quality of the reads with a sliding window of 4 bases and a minimum Phred score >20. Those quality-filtered reads were the input for the de novo transcriptome assembly with Trinity transcriptome assembler (version 2.5.1) [45], with the default parameters and the metadata file containing all the samples separated by condition evaluated. To validate the assembly, the reads of each sample were realigned to the transcriptome using Bowtie2 (version 2.3.0) [46]. The raw reads were deposited in the NCBI repository under the BioProject ID number PRJNA804904.

### 4.5. Functional Annotation

De novo assembled transcriptome was annotated using Trinotate pipeline (https://trinotate.github.io/ (accessed on 19 February 2021)) as reported in the axolotl transcriptome [47].

TransDecoder v2.0.1 was used to predict coding sequences and identify open reading frames (ORFs). The ORFs were scanned to search homology against the UniProt/SwissProt and UniRef90 databases using BLASTx and BLASTp (*e*-value < 1 × 10^−5^), respectively. Protein domains were identified using the Pfam domain database using HMMER v3.1b2 [48]. Potential signal peptides were identified using SignalP v4.1 [49] and transmembrane regions were predicted with TmMM [50] and rRNA with RNAmmer [51]. Moreover, assembled transcripts were also searched against the evolutionary genealogy of genes: Non-supervised Orthologous Groups (eggNog), Kyoto Encyclopedia of Genes and Genomes (KEGG), and Gene Ontology (GO) annotation databases. Transcriptome annotations were loaded into an SQLite database and reported in a tab-delimited file. Further, the trinotateR package was used to summarize the results of the transcriptome annotations. KEGG mapper pathway was used to identify the KO terms and then plot in Rstudio using the ggplot2 package.

### 4.6. Differential Expression and Functional Enrichment Analysis

The expression was calculated with RSEM (RNA-Seq by Expectation Maximization) (version 1.2.31) [52] filtering the features with an FPKM <1. The differential expression analysis was performed using the “run_DE_analysis.pl” script of the Trinity suite with the edgeR method [53]. Genes with a false discovery rate (FDR) cut-off *p* ≤ 0.001 and Log_2_FoldChange (LogFC) >2 were considered significant differentially expressed genes (DEG). Additionally, a subset annotation from the DEG was generated using the trinotateR package, and with this information, an online public database was created. The number of up and down-regulated genes was calculated by each pairwise comparison against Day 0. Then, Venn diagrams were generated to analyze the shared up and down-regulated genes by each temperature. Subsequently, genes associated with ripening were plotted in a heatmap with hierarchical clustering. These plots were carried out using the Venndiagram, pheatmap, and ggplot2 packages in Rstudio, respectively. Gene Ontology (GO) terms of DEG were performed in Rstudio using the GOseq package [54]. Indeed, GO terms related with ripening with a corrected *p*-value < 0.05 were considered significant, then enriched and summarized in Table 2. Then, KEGG functional enrichment analysis was performed with KOBAS [55] from the Entrez gene ID identified in the *Arabidopsis thaliana* database using the org.At.tair.db package in Rstudio. A scatter plot by each pairwise comparison against Day 0 of the top 10 GO terms and KEGG pathways was made using the ggplot2 package in Rstudio.

### 4.7. Correlation Analysis and Network Construction

To identify genes that show a significant association between the expression levels and firmness, correlation matrix between the GO enriched DEG related to cell wall and firmness was estimated by using Pearson correlation coefficient (r) and *p* < 0.05. Further, gene co-expression networks were constructed with the igraph package in Rstudio using the enriched DEG at 28 ± 2 °C and 15 ± 2 °C. Edges below r < 0.8 and vertices with no edges were removed.

### 4.8. Transcriptome Validation of Differential Expressed Genes by qRT-PCR

The previously extracted RNA was quantified using a NanoDrop ND-1000 UV-Vis spectrophotometer at 260 nm (Nano Drop Technologies Inc., Wilmington, DE, USA) and the integrity was analyzed by 1.5% agarose gel electrophoresis. Then, the first-strand cDNA was synthesized from 1 μg of total RNA using the SuperScriptIII reverse transcriptase kit according to the manufacturer’s instructions.

Primers and probe sequences of four selected genes were designed using the software Primer3 [56] as shown in Table 3. The specificity of the primers was tested by the Primer-Blast tool (http://www.ncbi.nlm.nih.gov/tools/primer-blast/ (accessed on 14 September 2021)). The qRT-PCR was carried out in a StepOnePlus™ Real-time PCR System (Applied Biosystems Inc, Foster City, CA, USA) with a final volume of 20 µL including 1X TaqMan Fast Advanced Master Mix (10 µL), 0.4 µM (0.8 µL) of forward and reverse primer, 0.18 µM (0.72 µL) of the probe, and 40 ng de cDNA (2 µL). Amplification conditions were one cycle of 95 °C for 5 min followed by 45 cycles of 95 °C for 1 min and 55 °C for 1 min with a signal acquisition in the FAM channel at the end of the annealing/extension step. Non-template controls were also included. Relative gene expression was calculated with the 2^−ΔΔCT^ method [57] using *Ubiquitin* (*UBC*) as a reference gene to normalize the data as previously reported by Berumen-Varela et al., 2020b, under the same conditions. Day 0 was considered the calibrator sample to calculate the final values. Gene expression values were reported as LogFC and plotted in Rstudio using the ggplot2 package.

### 4.9. Statistical Analysis

A randomized complete block design (days of storage as blocks) was used to analyze the physicochemical parameters (firmness, titratable acidity, TSS, and pH). On the other hand, LogFC for each gene was evaluated under a complete randomized design. Shapiro–Wilk test and Bartlett test were performed to all data to verify the normality and the homogeneity of variances, respectively. These data were analyzed by analysis of variance (ANOVA) with *p* < 0.05 significance level. Tukey’s HSD test (Honestly Significant Differences) was carried out when ANOVA showed significant differences. Linear regression and Pearson correlation analysis were done to validate the LogFC gene expression values obtained by qRT-PCR and RNA-seq results. Plots and statistical analysis were performed in RStudio using the ggpubr, ggplot2, and agricolae packages.

## 5. Conclusions

The temperature of 15 ± 2 °C is a promising strategy and viable technology to transport soursop fruits long distances and reach national and international markets, leading to an increase in the income for the Mexican producers. The gene expression of soursop fruits is regulated by the temperature and days of postharvest storage. This study provides valuable information to establish the molecular basis to start a germplasm bank and breeding program to develop a soursop variety with longer postharvest shelf life.

## Figures and Tables

**Table 1:** Number of unique and total functional annotation of the soursop fruit transcriptome using Trinotate pipeline.

Annotation Results	Unique Number of Sequences	Total Number of Sequences
Protein hits (BLASTx-Uniref90)	132,770	5,598,731
Protein hits (BLASTx-Uniprot/Swissprot) sprot_Top_BLASTX_hit	104,280	4,673,452
GO (BLASTx) gene_ontology_BLASTX	11,829	4,483,769
eggNOG	5699	4,042,793
KGG	18,287	3,902,577
TmHMM	81,118	3,55,812
Protein hits (BLASTp-Uniref90)	136,059	2,31,177
Protein hits (BLASTp-Uniprot/Swissprot) sprot_Top_BLASTP_hit	94,538	1,55,333
GO (BLASTp) gene_ontology_BLASTP	13,307	149,975
Pfam	77,368	125,655
GO Pfam gene_ontology_Pfam	2388	78,880
SignalP	5579	47,554
RNAMMER	30	442

**Table 2:** Enriched GO terms related to the cell wall, pectin, carbohydrates (polysaccharide and disaccharides), and glycosylation.

GO ID	GO Annotation	Corrected *p*-Value				
		28 °C ± 2 °C		15 °C ± 2 °C		
		Day 3	Day 6	Day 3	Day 6	Day 9
GO:0042545	cell wall modification	1.28 × 10^−10^	1.33 × 10^−11^	2.71 × 10^−8^	6.42 × 10^−3^	5.65 × 10^−4^
GO:0071555	cell wall organization	1.45 × 10^−10^	2.03 × 10^−11^	2.20 × 10^−7^	1.48 × 10^−2^	1.93 × 10^−3^
GO:0071554	cell wall organization or biogenesis	4.52 × 10^−10^	7.37 × 10^−11^	4.89 × 10^−7^	2.12 × 10^−2^	3.213 × 10^−3^
GO:0005618	cell wall	5.67 × 10^−10^	7.05 × 10^−10^	1.025 × 10^−7^	5.44 × 10^−4^	1.83 × 10^−4^
GO:0030599	pectinesterase activity	1.28 × 10^−10^	1.33 × 10^−11^	2.71 × 10^−8^	6.42 × 10^−3^	5.65 × 10^−4^
GO:0004650	polygalacturonase activity	2.45 × 10^−8^	1.81 × 10^−12^	2.88 × 10^−7^	6.40 × 10^−6^	4.24 × 10^−7^
GO:0000272	polysaccharide catabolic process	1.03 × 10^−5^	2.33 × 10^−5^	3.42 × 10^−6^	4.50 × 10^−5^	3.06 × 10^−5^
GO:0005976	polysaccharide catabolic process	1.26 × 10^−3^	5.58 × 10^−5^	2.57 × 10^−4^	2.89 × 10^−5^	5.45 × 10^−4^
GO:0046351	disaccharide biosynthetic process	3.00 × 10^−4^	3.71 × 10^−6^	5.30 × 10^−5^	4.26 × 10^−4^	5.25 × 10^−4^
GO:0005984	disaccharide metabolic process	3.35 × 10^−5^	2.66 × 10^−9^	6.99 × 10^−4^	2.53 × 10^−5^	3.13 × 10^−5^
GO:0004553	hydrolase activity, hydrolyzing O-glycosyl compounds	5.49 × 10^−24^	1.25 × 10^−30^	3.39 × 10^−15^	3.99 × 10^−16^	3.36 × 10^−15^
GO:0016798	hydrolase activity, acting on glycosyl bonds	3.91 × 10^−23^	1.19 × 10^−29^	9.95 × 10^−15^	3.40 × 10^−15^	2.69 × 10^−14^

**Table 3:** Primer’s sequence used to amplify the genes of this study. Fw and Rv mean forward and reverse primers, respectively.

Gene Name	Sequence (5′–3′)	Amplicon Size (bp)
***EDR1***	Fw: TTTTGGCAGACAGTGTGGGT	
	Rv: TCAGATGGGATAAGCGTGCC	151
	Probe: GGTTGATCAAAGGGCAGCAA	
***EXP4***	Fw: GAGGACGGATTGGATGGCTA	
	Rv: TCGGAAGGAGAGAGACTGGG	88
	Probe: CAGAACAGGCAGTCGAACG	
***PL15***	Fw: GGACAATGGCTGACGGTGAT	
	Rv: TGCATCTACAAGGCCATCGG	101
	Probe: ATCACTGCTCCCTCTCCAAC	
***PME2***	Fw: GCCGGTCTCTCCCTGTAAAC	
	Rv: TAAGGCTCCATCCGAATCGC	80
	Probe: CATGTAGGATGCCATTGCCA	

## Data Availability

All data are available upon reasonable request.

## Supplementary Materials

The following supporting information can be downloaded at: https://www.mdpi.com/article/10.3390/plants11141798/s1, Table S1: LogFC values obtained by RNA-Seq and qRT-PCR.
