# Supplementary material for: Transcriptome Analysis of Soursop (Annona muricata L.) Fruit under Postharvest Storage Identifies Genes Families Involved in Ripening
Source: Plants (Basel). 2022 Jul 7;11(14):1798. doi: 10.3390/plants11141798 (PMC9325311; doi:10.3390/plants11141798)
Supplement: Supplementary file 1 [file plants-11-01798-s001.zip › plants-1792975-supplementary Table S1.pdf]

Table S1. LogFC values obtained by RNA-Seq and qRT-PCR

Gene	Treatment	LogFC by RNA-seq	LogFC by RT-qPCR
EDR1	Day 3 at 28±2°C	-2.31	-1.17
	Day 6 at 28±2°C	-1.63	-0.89
	Day 3 at 15±2°C	-0.8	-0.38
	Day 6 at 15±2°C	-2.56	-2.05
	Day 9 at 15±2°C	-2.35	-2.11
EXP4	Day 3 at 28±2°C	6.14	3.10
	Day 6 at 28±2°C	3.64	3.05
	Day 3 at 15±2°C	7.19	2.46
	Day 6 at 15±2°C	8.4	2.94
	Day 9 at 15±2°C	8.22	2.96
PL15	Day 3 at 28±2°C	3.22	2.03
	Day 6 at 28±2°C	1.32	1.42
	Day 3 at 15±2°C	8.11	2.08
	Day 6 at 15±2°C	6.61	2.25
	Day 9 at 15±2°C	6.02	2.43
PME2	Day 3 at 28±2°C	-3.54	0.11
	Day 6 at 28±2°C	-2.4	0.71
	Day 3 at 15±2°C	-0.73	-0.50
	Day 6 at 15±2°C	-2.18	-0.21
	Day 9 at 15±2°C	-2.45	0.40
